# Serum vaspin concentration in elderly patients with type 2 diabetes mellitus and macrovascular complications

**DOI:** 10.1186/s12902-017-0216-0

**Published:** 2017-10-24

**Authors:** Wei Yang, Yun Li, Tian Tian, Li Wang, Pearl Lee, Qi Hua

**Affiliations:** 1 Department of Geriatric Medicine, Capital Medical University, Xuan Wu Hospital, Beijing, 100053 China; 2 Department of Endocrine, Capital Medical University, Xuan Wu Hospital, Beijing, 100053 China; 3 Divisions of Geriatric and Palliative Care Medicine, University of Michigan, Ann Arbor, MI 48109 USA; 4 Department of Cardiology, Xuanwu Hospital, Capital Medical University, No. 45 Changchun Street, Beijing, 100053 China

**Keywords:** Vaspin, Adipokines, Type-2 diabetes mellitus, Elderly, Macrovascular complications

## Abstract

**Background:**

Adipose tissue, an endocrine organ of the body, is involved in some obesity-related disease states such as insulin resistance, diabetes mellitus, and atherosclerosis. Vaspin is a novel adipocyte with insulin sensitizing effects. In this study, we planned to estimate serum vaspin concentrations as related to glycemic status and the presence of macrovascular complications among elderly patients with type-2 diabetes mellitus (T2DM).

**Methods:**

A total of 230 elderly patients with T2DM were evaluated. These patients were divided into two groups: patients without complications (T2DM group, *n* = 110), and patients with macrovascular complications (T2DM + MC group, *n* = 120). In addition, 60 healthy elderly subjects were enrolled and assigned into the control group (NC group). Relevant parameters were matched for age and gender ratio. Serum vaspin concentrations were measured by Enzyme-linked immunosorbent assay (ELISA). Anthropometric measurements, plasma glucose and HbA_1C_ levels, insulin concentration, liver and kidney functions, and lipid profile were measured for each participant.

**Results:**

Serum vaspin concentrations were significantly higher in the T2DM group than in the T2DM + MC group (*F* = 13.122, *P* < 0.01). These concentrations were also significantly higher among females, compared to males (*T* = 3.567, *P* < 0.05). Logistic regression analysis revealed that serum vaspin concentration, systolic blood pressure, HDL-C and T2DM duration were independent influencing factors for diabetic macrovascular complications.

**Conclusion:**

Serum vaspin may be considered as a potential marker to assess the status of elderly patients with T2DM and the risk of developing serious macrovascular complications. Further prospective studies are warranted.

**Trial registration:**

ChiCTR-OPC-14005698, retrospectively registered on 20 Dec. 2014.

## Background

With over 400 million people having diabetes worldwide, a rapid increase in the prevalence of diabetes has been found in both developed and developing countries [1]. The latest epidemiological study shows that China has a higher prevalence rate, compared to the United States [2]. The prevalence of type-2 diabetes mellitus (T2DM) in a ≥ 18-year-old and ≥60-year-old Chinese population is 9.7% and 19.6%, respectively [3, 4]. Furthermore, its incidence, severity, disability and mortality rates are higher in elderly patients with T2DM [5]. T2DM is a well-established risk factor for macrovascular complications. It is associated with significantly increased risk of ischemic stroke, heart failure and non-fatal myocardial infarction [6]. Macrovascular disease is a major cause of death and a leading cause of disability and the impaired quality of life of older adults with diabetes [7]. Furthermore, T2DM in the elderly has placed a heavy burden on society and in the economic development of the country [8]. Thus, it is important to understand preventive strategies to control the incidence of T2DM, and simultaneously explore the different mechanism of development and progression of macrovascular diseases.

Adipose tissue is responsible for energy storage, and has been found to secrete a variety of biologically active substances such as leptin, resistin, adiponectin, interleukin-6, tumor necrosis factor and visfatin, which are collectively referred to as adipocytokines [6–9]. Furthermore, adipose tissue is involved in regulating insulin levels, and is associated with T2DM [7].

Visceral adipose tissue-derived serine protease inhibitor (vaspin) is a novel candidate that links human obesity and its related metabolic alterations. Several animal studies have implied an association between vaspin and the severity of T2DM. Vaspin was isolated from Otsuka Long-Evans Tokushima Fatty (OLETF) T2DM rats by Japanese researchers in 2005, and subsequent animal experiments revealed its insulin sensitizing effect [10]. Hida et al. [4] found that vaspin concentrations decreased with the worsening of T2DM in OLETF rats. Furthermore, vaspin expression was shown to decrease with the worsening of T2DM and loss of body weight, while insulin or pioglitazone treatment helped in achieving normal serum concentrations of vaspin [11]. Additionally, the administration of vaspin to obese mice improved glucose tolerance and insulin sensitivity, and altered the gene expression of candidate genes for insulin resistance [12].

As for clinical research, a number of previously conducted clinical trials have shown that vaspin, obesity, glucose metabolism, T2DM and insulin resistance are closely related [4, 10–13]. Previous studies [4, 10–14] have indicated that the concentration of vaspin may be associated to the occurrence and development of atherosclerosis among diabetic patients [15]. However, few studies have been conducted among the elderly population. In addition, less focus was given on the relationship between vaspin and diabetic vascular disease. We aimed to evaluate the association between serum vaspin concentrations, glycemic status and the presence of macrovascular complications among elderly patients with T2DM.

## Methods

### Patient selection

This cross-sectional observational study enrolled a total of 230 patients (> 60 years old) diagnosed with T2DM from July 2014 to January 2016. All participants provided a written informed consent before taking part in the study. Additionally, subjects in the NC group (37 males and 23 females, mean age: 73.3 ± 6.2 years) were selected from of Department of Geriatrics and Endocrinology of Xuanwu Hospital, which served as controls.

Diabetic macrovascular disease was diagnosed on the basis of the degree of carotid artery disease. If the carotid ultrasound revealed stenosis or occlusion, it was considered to represent the presence of macrovascular disease. The 230 elderly patients were divided into two groups, according to the presence or absence of macrovascular disease: T2DM group, 110 cases (73 males and 39 females, mean age: 75.1 ± 2.1 years); T2DM + MC group, 120 cases (77 males and 41 females, mean age: 76.1 ± 3.1 years).

Healthy controls were recruited from the clinic of the Geriatric and Endocrine Department. All participants underwent the OGTT test, and had confirmed normal glucose tolerance.

### Inclusion criteria

Patients > 60 years and those who satisfied the standard diagnostic criteria of T2DM were included into the study. T2DM was diagnosed when one of these three criteria was met: (1) fasting plasma glucose (FPG) **≥ **126 mg/dL, (2) symptoms of hyperglycemia and random fasting glucose ≥ 200 mg/dL, or (3) two-hour plasma glucose ≥ 200 mg/dL. Those who satisfied the standard diagnostic criteria for T2DM with macrovascular complications (China Diabetes Guidelines [2013]) were enrolled into the study [16].

### Exclusion criteria

Patients were excluded based on the following criteria: patients with type-1 diabetes mellitus; patients with acute complications of diabetes mellitus; patients who have severe infections; patients with a history of tumor; patients who have other endocrine or autoimmune diseases, or patients who have been using hormonal preparations or immune inhibitors such as hydrocortisone, prednisone, methylprednisolone, cyclosporine A, and/or tacrolimus for more than 1 week.

### Sample size calculation

The sample size was estimated to ensure sufficient power in the analysis of variance. Based on a previous study [13–15], the mean (standard deviation) of vaspin in the control, TM and TM + MC groups were 368.5 (11.8), 572.4 (22.4) and 167.4 (25.4), respectively. In order to achieve 90% power with a significance level of 0.05, 62 participants in each group were needed.

### Anthropometric measurements

The weight (Kg) and height (cm) of the enrolled subjects were recorded. Body mass index (BMI) was determined by dividing the weight (Kg) by the height (m^2^). Measurement of waist circumference (cm): This was measured just above the uppermost lateral border of the right iliac crest. A horizontal mark was drawn, and crossed with a vertical mark on the mid-axillary line.

The measuring tape was placed in a horizontal plane around the abdomen at the level of this marked point on the right side of the trunk.

### Parameters for clinical assessment

Blood samples were obtained from all participants between 8 and 10 A.M. after a 12-h overnight fast, and the samples were immediately centrifuged. Aliquots of serum and plasma were taken to analyze the biochemical markers studied. Serum samples were stored at −80 °C until measurements of vaspin and insulin levels.

Blood glucose including fasting blood glucose (FBG) and postprandial two-hour glucose (2-h PBG) (after 75 g of oral glucose load) was measured using a glucose oxidase procedure. Glycosylated hemoglobin (HbA1c%) was measured using a Cobas Integra 800 automated biochemistry analyzer (Roche, Basel, Switzerland).

Fasting serum insulin (FSI) concentration was measured using the radioimmunoassay analysis method (Ray Bio, Norcross, GA). High-sensitivity C-reactive protein (hs-CRP) was detected by enzyme-linked immunosorbent assay (ELISA), and lipid-related biochemical indicators such as total cholesterol (TC), triglyceride (TG), low-density lipoprotein (LDL-C) and high-density lipoprotein (HLD-C) were measured using a Hitachi 7600 automatic biochemical analyzer. All these were performed at the same time and place. Insulin resistance index (homeostasis model assessment of insulin resistance, HOMA-IR) was measured using the equation: HOMA-IR = FPG (mmol/L) × FINS (μU/L) / 22.5.

Based on the following formula: coefficients of variation (CV) = (standard deviation / mean) × 100%; and the inter- and intra-CV for insulin is 10% and 7%, respectively.

### Measurement of vaspin

A portion of each blood sample was separated to obtain the serum by centrifugation at 3500 rpm for 5 minutes, and these were stored at −80 °C. The serum vaspin concentration was measured by ELISA in a fully automatic multifunctional enzyme standard instrument (Thermo USA). The inter- and intra-CV for vaspin is 8% and 6% respectively.

### Statistical analysis

Data was analyzed using SPSS for Windows, Version 18.0 (SPSS Inc., Chicago, IL, USA). Data were presented as mean ± standard error of the mean (SEM). Before performing the statistical analysis, parameters without a normal distribution were logarithmically transformed to approximate the normal distribution. Single factor analysis of variance was used to compare measurement indexes among multiple groups. The *c*2-test or Fisher’s exact test was used for the analysis of categorical variables. Logistic regression analysis was used in the multivariate analysis. A *P*-value of 0.05 was considered statistically significant.

## Results

### Clinical characteristics of subjects

A total of 150 males and 80 females with a mean age of 75.7 ± 3.3 years were included into this study. The clinical characteristics of patients in the T2DM and T2DM + MC groups, and in subjects in the control group, are shown in Table 1.

**Table 1:** Clinical characteristics of the three study groups

	T2DM	T2DM + MC	Control
Gender (M/F)	73/39	77/41	37/23
Age (years)	75.1 ± 2.1	76.1 ± 3.1	73.3 ± 6.2
Duration (years)	15.2 ± 4.3	16.8 ± 3.4	NA
BMI (kg/m^2^)	26.17 ± 3.83^★^	27.10 ± 4.01^★^	23.13 ± 3.83
WHR	0.94 ± 0.63^★^	0.98 ± 0.31^★^	0.85 ± 0.07
SBP (mmHg)	137 ± 9^★^	143 ± 13^★^	123 ± 16
DBP (mmHg)	69 ± 6	67 ± 6	65 ± 7
AF, *n*	34	45	12
CKD, *n*	33^★^	45^★^	10
CHD, *n*	37^★^	55^★●^	16
1	64	45	None
2	33	47	None
3	13	28^**●**^	None

Data are presented as mean ± standard error of the mean (SEM)

*NS* not significant, *NA* not applicable, *DM* diabetes mellitus, *BMI* body mass index, *WHR* Waist to hip ratio, *SBP* systolic blood pressure, *DBP* diastolic blood pressure, *AF* atrial fibrillation, *CKD* Chronic kidney disease, *CHD* coronary heart disease

Compared with the T2DM group: ^●^ *P* < 0.05; Compared with the control group: ^★^ *P* < 0.05

Age, gender distribution, duration of diabetes and diastolic blood pressure (DBP) were similar in these three subgroups. However, BMI, waist-hip ratio (WHR), systolic blood pressure (SBP) and co-morbidities such as chronic kidney disease (CKD) and coronary heart disease (CHD) were significantly higher in patients in the T2DM group than in subjects in the control group (*P* < 0.05). These parameters and disease conditions were similar between the T2DM and T2DM + MC groups (*P* < 0.05). The number of patients using three anti-diabetic drugs in the T2DM + MC group was significantly higher than in the T2DM group (*P* < 0.05). Furthermore, the use of one or two anti-diabetic drugs was similar between these two groups.

### Biochemical characteristics of subjects

Concentrations of FPG, plasma insulin, HOMA-β, HbA_1c_, hs-CRP and HDL-C were significantly higher among patients in the T2DM group than in subjects in the control group (*P* < 0.05) (Table 2). The concentration of total cholesterol, LDL-C and triglycerides were similar in these three groups.

**Table 2:** Biochemical properties of the objectives of the three groups

	T2DM	T2DM + MC	Control
FPG (mg/dl)	9.42 ± 4.20^**★**^	8.76 ± 3.16^**★**^	5.11 ± 1.20
Insulin (mIU/ml)	24.7 ± 81.7^**★**^	22.8 ± 49.1^**★**^	9.7 ± 10.2
HOMA-IR	7.14 ± 21.18^**★**^	9.97 ± 21.64^**★●**^	3.41 ± 2.72
HbA_1c_ (%)	8.07 ± 1.97^**★**^	8.55 ± 2.17^**★**^	5.58 ± 0.41
TC (mg/dl)	4.29 ± 2.12	4.35 ± 0.87	4.01 ± 1.03
LDL-C (mg/dl)	2.41 ± 0.75	2.39 ± 1.17	2.62 ± 0.93
HDL-C (mg/dl)	1.18 ± 0.45^**★**^	1.03 ± 0.50^**★**^	1.79 ± 0.59
TG (mg/dl)	1.74 ± 1.24	1.81 ± 1.41	1.72 ± 0.69
hs-CRP (mg/dl)	5.15 ± 4.30^**★**^	6.02 ± 6.86^**★**^	2.31 ± 3.84
Vaspin (ng/ml)	592.5 ± 45.2^**★**^	177.6 ± 54.8^▲**●**^	381.9 ± 32.6

*FPG* fasting plasma glucose, *HOMA-IR* homeostasis model assessment of insulin resistance, *HbA1c* glycosylated hemoglobin A1C, *TC* total cholesterol, *LDL-C* low density lipoprotein-cholesterol, *HDL-C* high-density lipoprotein cholesterol, *TG* triglycerides, *hs-CRP* C-reactive protein

Compared with the T2DM group: ^●^ *P <* 0.05; Compared with the control group: ^★^ *P* < 0.05, ^▲^ *P* < 0.01

The concentration of HOMA-IR was highest in the T2DM + MC group (9.97 ± 21.64, *P* = 0.013). Compared with the other two groups, the concentrations were significantly higher in the T2DM + MC group (*P* < 0.05, for both).

### Vaspin levels

**Fig. 1:**
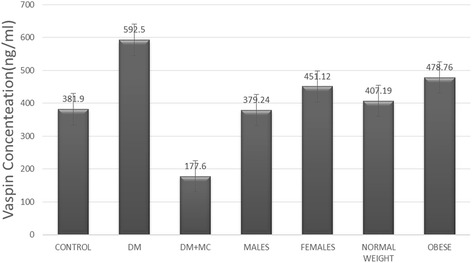
Serum vaspin concentrations of the different subgroups

There were significant differences in serum vaspin concentrations among the three groups (F = 14.76, *P* < 0.01). Serum vaspin concentration was significantly higher in the T2DM group than in the control group (*P* < 0.05), significantly lower in T2DM + MC group than in the T2DM group (*P* < 0.01), and significantly lower in the T2DM + MC group than in the control group (*P* < 0.05). Serum vaspin concentration in females was significantly higher than males (451.12 ± 24.21 and 379.24 ± 18.32, respectively; *P* = 0.25). In addition, serum vaspin concentration was significantly higher among obese patients, when compared to patients with normal weight (478.76 ± 32.35 and 407.19 ± 13.21, respectively; *P* = 0.32) (Fig. 1).

### Correlation analysis

Significant positive correlations between vaspin and TG levels (*r* = 0.36, *P* = 0.046), FINS (*r* = 0.56, *P* = 0.032) and HOMA-IR (*r* = 0.78, *P* = 0.016) were found among elderly patients with T2DM (Table 3). Vaspin concentrations did not correlate with glycemic measurements including FPG and HbA_1c_ in either the control group, T2DM group, or T2DM + MC group.

**Table 3:** Pearson correlation of vaspin with other indices

	Control group		T2DM and T2DM + MC groups	
	Correlation coefficient	*P*-value	Correlation coefficient	*P*-value
Age	0.07	0.654	0.02	0.789
BMI	0.12	0.564	0.05	0.685
WHR	0.18	0.512	0.11	0.112
TC	0.23	0.487	0.13	0.098
TG	0.41	0.054	0.36	0.046
LDL-C	0.23	0.487	0.21	0.073
HDL-C	0.65	0.034	0.16	0.086
HOMA-IR	0.78	0.028	0.78	0.016
FPG	0.21	0.496	0.22	0.069
FINS	0.33	0.075	0.56	0.032
HbA1c	0.27	0.308	0.13	0.011

*BMI* body mass index, *WHR* Waist to hip ratio, *TC* total cholesterol, *TG* triglycerides, *LDL-C* low density lipoprotein-cholesterol, *HDL-C* high-density lipoprotein cholesterol, *HOMA-IR* homeostasis model assessment of insulin resistance, *FPG* fasting plasma glucose, *HbA1c* glycosylated hemoglobin A1C

### Multivariate logistic regression analysis

Multivariate logistic regression analysis was applied to data obtained from all T2DM patients. With macrovascular disease as the dependent variable, and gender, age, duration of diabetes, BMI, WHR, SBP, vaspin, hsCRP, TG, TC, LDL-C, HDL-C, FPG, FINS, HOMA-IR and HbA_1C_ as independent variables, the analysis revealed a significant positive association between the presence of macrovascular disease and SBP and the duration of diabetes and HOMA-IR (*P* = 0.032, 0.045, 0.016, respectively), and a significant inverse association between the presence of macrovascular disease and HDL-C (*P* = 0.034) and serum vaspin concentration (*P* = 0.024) (Table 4).

**Table 4:** Logistic regression analysis results of risk factors for the T2DM and T2DM + MC groups

	OR	95% CI	*P*-value
DURATION	1.782	1.543-1.935	0.032
SBP	1.345	1.012-1.532	0.045
VASPIN	0.734	0.512-0.912	0.024
HOMA-IR	1.543	1.212-1.754	0.016
HDL-C	0.625	0.435-0.912	0.034

*SBP* systolic blood pressure, *HOMA-IR* homeostasis model assessment of insulin resistance, *HDL-C* high-density lipoprotein cholesterol

## Discussion

### Summary of the study results

In the present study, we found that the serum concentration of vaspin was highest in the T2DM group and lowest in the T2DM + MC group. There was a significantly lower level of serum vaspin concentration in the T2DM + MC group than in the control group (*P* < 0.05). Logistic regression analysis revealed that serum vaspin concentrations were independent influencing factors of diabetic macroangiopathy (OR: 0.734, 95% CI: 0.512-0.912). HOMA-IR concentration was highest in the T2DM + MC group, as compared to the other two groups. Correlation analysis revealed a positive correlation between serum vaspin concentrations and HOMA-IR (*r* = 0.78, *P* < 0.05) in both diabetic patients and subjects in the control group.

### Association between serum vaspin concentration and glucose metabolism in the elderly

In the present study, serum vaspin concentration was significantly higher in the T2DM group, compared to the control group (*P* < 0.05). These results were similar to some former studies, which indicated that vaspin is associated with glucose metabolism. Li et al. found that fasting serum vaspin concentration in diabetic patients (with or without plaque) was also significantly higher than in controls (*P* < 0.05) [11]. Gulcelik et al. [17] identified that diabetic women with good glycemic control had lower concentrations of vaspin than diabetic women with poor glycemic control. However, other trials reported contradictory results. Seeger et al. [18] failed to find a correlation between vaspin concentrations and glucose metabolism, including fasting glucose and FINS, in diabetic patients with hemodialysis. Youn et al. [19] found that serum vaspin levels were not different between males with normal glucose tolerance (NGT) and those with T2DM. Furthermore, the concentration of vaspin was not significantly higher (1.5-fold) in female subjects with NGT as compared to female subjects with T2DM (*P* = 0.085). This inconsistency may be attributed to patient characteristics and treatments. As shown in the present study, the concentration of vaspin may be affected by TG, LDL-C and HbA_1_C, which are also factors closely related to diabetes.

### Relationship between vaspin and T2DM with macrovascular disease in the elderly

It has been thought that vaspin may regulate glucose metabolism, improve insulin resistance, inhibit inflammatory reactions and delay the progress of atherosclerosis [7]. The findings in the present study are consistent with most clinical results among adult patients. One study on elderly diabetic twins revealed that serum vaspin concentration decreased with the development of the emergence of diabetic complications [20]. Li et al. has shown that the concentration of vaspin revealed a downward trend with the progression of diabetes and the appearance of large vascular lesions [11]. Jian et al. studied patients with T2DM for 2 years, and found that low serum concentration of vaspin is a risk factor for the progression of T2DM [21]. Another study also revealed that serum vaspin concentration decreased as an independent risk factor for T2DM and carotid atherosclerosis [11]. Furthermore, a trial found that microvascular complications among patients with T2DM on metformin therapy had low concentrations of serum vaspin than prior treatment [17]. It is noted that compared with younger adult patients (age < 60 year old) in previous studies [11, 15], we found that the patients in our study were older, and that there was a significant reduction in vaspin among them, suggesting that the reverse association between vaspin concentration and macrovascular disease among diabetic patients may be more significant in the elderly. However, there is a lack of convincing evidence on whether anti-diabetic medication can decrease the level of vaspin in diabetic patients with macrovascular complications.

### The relationship between vaspin and insulin and insulin resistance in elderly patients

Our study suggests a positive correlation between serum vaspin concentrations and HOMA-IR. This correlation was also found in a study, which included a larger number of elderly patients [17]. Klöting et al. found vaspin mRNA levels among overweight or obese subjects [22]. These levels were found to be expressed in visceral or subcutaneous adipose tissues, and this expression in subcutaneous adipose tissues was positively correlated with FINS, and negatively correlated with the insulin sensitivity index. Such positive correlation of serum vaspin concentration with FINS and HOMA-IR was even found in obese children [4]. Moreover, in type-2 recent-onset diabetic patients, elevated plasma vaspin levels can be decreased after continuous subcutaneous insulin infusion treatment [23]. Rosiglitazone therapy for patients with poor glycemic control appears to decrease plasma vaspin levels through glucose and insulin sensitivity regulation [24]. These results imply that insulin resistance is probably associated with high concentrations of vaspin, and anti-diabetic medication can probably decrease the level of vaspin in new-onset patients. However, the relationship between vaspin and insulin concentrations and HOMA-IR remains controversial at this stage. Another study revealed that there was no correlation between vaspin concentration and HOMA-IR [25, 26]. Further studies are warranted to clarify this association.

### Strength and limitations

This study added evidence for the association of serum vaspin with insulin resistance, T2DM and its macrovascular complication, since substantial uncertainty about this association remains in previous studies. We included elderly patients >70 years old, who were tended to be excluded in a previous research.

However, some limitations should be mentioned and taken into account for the interpretation of the study results. First, as a cross-sectional study, this study could not confirm the causal effects of serum vaspin on the development of T2DM and its macrovascular complications. Furthermore, a reverse causal association could not be precluded. However, the association of serum vaspin with T2DM and its macrovascular complications observed in the present study may help generate a hypothesis that vaspin may be involved in the pathogenesis of T2DM and macrovascular complications. Second, some factors, such as dietary habits and lifestyle behavior, are likely associated with serum vaspin, T2DM and macrovascular complications. These factors were not measured in our study, which may lead to residual confounding. Third, we only included eligible patients from a single hospital. Therefore, the generalization of findings to other populations should be given considerable caution.

### Implication for clinical practice

Vaspin may have a compensatory effect on impaired glucose metabolism, insulin resistance, or inflammatory reactions in elderly patients at the initial stage of the disease, such as before the development of macrovascular complications. Serum vaspin may be considered as a potential marker to evaluate the progression of T2DM and the development of macrovascular complications.

### Implication for future research

Our study generates a hypothesis that serum vaspin may play an important role in insulin resistance, T2DM and macrovascular complications. Further investigations are needed to understand the regulation of vaspin and its role in the development and course of T2DM in the elderly. Given its cross-sectional design, this study alone cannot possibly establish a causal relationship. Further prospective studies are warranted to confirm all the observed associations in our study.

## Conclusions

In conclusion, serum vaspin appears to play an important role in insulin resistance, T2DM and macrovascular complications. Further prospective studies are warranted to provide more confirmatory evidence.

## Abbreviations

BMI: Body mass index
CHD: Coronary heart disease
CKD: Chronic kidney disease
DBP: Diastolic blood pressure
DM: Diabetes mellitus
ELISA: Enzyme Linked Immunosorbent Assay
FINS: Fasting insulin
FPG: Fasting plasma glucose
HbA1C: Glycosylated hemoglobin
HDL-C: High-density lipoprotein cholesterol
HOMA-IR: Homeostasis model assessment of insulin resistance
Hs-CRP: High-sensitivity C-reactive protein
LDL-C: Low density lipoprotein
MC: Macro-vascular complications
NC: Non-complications control
OLETF: Otsuka Long-Evans Tokushima Fatty
SBP: Systolic blood pressure
SEM: Standard error of the mean
T2DM: Type 2 diabetes mellitus
TC: Total cholesterol
TG: Triglyceride
WHR: Waist hip ratio
